# Extremitätenerhaltende Resektion von Weichteilsarkomen in der Regio axillaris

**DOI:** 10.1007/s00064-023-00824-8

**Published:** 2023-07-18

**Authors:** Ricarda Stauss, Tilman Graulich, Tarek Omar Pacha, Mohamed Omar

**Affiliations:** 1 Klinik für Unfallchirurgie, Medizinische Hochschule Hannover, Carl-Neuberg-Str. 1, 30625 Hannover, Deutschland; 2 Klinik für Unfallchirurgie, Sarkom-Zentrum, Medizinische Hochschule Hannover, Carl-Neuberg-Str. 1, 30625 Hannover, Deutschland

**Keywords:** Weichgewebesarkome, Muskuloskelettale Tumore, Extremitätenerhalt, Axilla, Onkologische Resektionen, Soft tissue sarcoma, Musculosceletal tumor, Limb salvage, Axillary space exploration, Oncological resections

## Abstract

**Operationsziel:**

Tumoren mit Lokalisation in der Regio axillaris stellen durch die enge anatomische Beziehung zu neurovaskulären Strukturen eine besondere Herausforderung für die Resektion dar. Operationsziel ist die R0-Resektion unter Wahrung eines maximalen Funktionserhalts.

**Indikationen:**

Weichteilsarkome, Metastasen.

**Kontraindikationen:**

Fortgeschrittene Stadien mit Infiltration neurovaskulärer Strukturen, Lokalisation des Biopsietrakts erfordert ausgedehnte Resektion, palliative Behandlungssituation.

**Operationstechnik:**

Erweiterter deltoideopektoraler Zugang. Ansatznahes Ablösen des M. pectoralis major et minor. Präparation der Vasa axillaria und der Faszikel des Plexus brachialis. Ligatur der in den Tumor eintretenden Gefäße. Tumorresektion, Fadenmarkierung. Rekonstruktion mittels transossärer Reinsertion des M. pectoralis minor am Processus coracoideus. Setzen von Bohrkanälen und transossäre Refixation des M. pectoralis major am Humerus.

**Weiterbehandlung:**

Schulterabduktionskissen für 6 Wochen, passive Mobilisation Woche 6 bis 12, dann aktive Mobilisation. Kompressionsorthese. Onkologische Nachsorge.

**Ergebnisse:**

Zwischen 2017 und 2022 erfolgte in 6 konsekutiven Fällen (4 primäre Weichteilsarkome, 2 Metastasen) die chirurgische Resektion. In 100 % der Fälle wurde eine primäre R0-Resektion erreicht. Das mittlere Follow-up lag bei 22,5 Monaten (3 bis 60 Monate), im gesamten Nachuntersuchungszeitraum traten keine Lokalrezidive auf. Der durchschnittliche Bewegungsumfang für die Abduktion im Schultergelenk lag bei 135,0 ± 41,4° (Range 90–180°). Es traten keine postoperativen sensomotorischen Defizite auf. Die subjektive Schulterfunktion lag bei 80,0 ± 21,0 % (Range 50–100 %). Der mittlere MSTS-Score lag bei 89,5 % (Range 32–100 %) und belegt somit ein gutes funktionales Outcome.

## Abstract (en)

**Objective:**

For patients with soft tissue sarcoma, surgical resection is a key element of curative therapy. Surgery is performed as a wide resection with microscopically negative margins (R0 resection) and as limb-sparing procedure whenever possible to preserve maximum function.

**Indications:**

Soft tissue sarcoma, metastases.

**Contraindications:**

Extensive disease with major neurovascular involvement, placement of biopsy tract necessitates extensive resection, palliative care.

**Surgical technique:**

Extended deltopectoral approach. Release of pectoralis major and minor tendons. Vascular and neurologic exploration, identification of the axillary vessels and brachial plexus, placing of loops around major structures. Mobilization of these structures to achieve adequate exposure. Clipping of vessels entering the tumor. Tumor resection, suture marking for histological analysis. Soft tissue reconstruction by transosseous reinsertion of the pectoralis minor to the coracoid process. Drill channel placement, transosseous refixation of the pectoralis major to the humerus.

**Postoperative management:**

Shoulder abduction brace for 6 weeks, passive mobilization for 6–12 weeks followed by active mobilization. Compression sleeve. Oncological follow-up.

**Results:**

Between 2017 and 2022, wide resection was performed in 6 consecutive cases including 4 primary soft tissue sarcomas and 2 metastases. Primary R0 resection was achieved in 100%. Mean follow-up was 22.5 months (3–60 months). There were no local recurrences. Mean active shoulder abduction was 135.0 ± 41.4° (90–180°). Neurological deficits were not observed. Mean subjective shoulder function was 80.0 ± 21.0% (50–100%). The mean Musculoskeletal Tumor Society (MSTS) score was 89.5% (32–100%), indicating good functional outcome in the study cohort.

## Vorbemerkungen

### Weichgewebesarkome in der Regio axillaris

Raumforderungen der Axilla umfassen primäre Weichgewebesarkome sowie hämatogene und lymphogene Metastasen unterschiedlicher Tumoren. Weichgewebesarkome sind eine heterogene Gruppe verschiedener Tumorentitäten mesenchymalen Ursprungs und machen etwa 1 % der malignen Neoplasien des Erwachsenen sowie 7–15 % der malignen Neoplasien bei Kindern aus [1–3]. Derzeit sind mehr als 50 histologische Subtypen bekannt [4]. Prinzipiell können Weichteilsarkome an jeder anatomischen Lokalisation auftreten. In absteigender Häufigkeit betrifft dies die Extremitäten (59 %), den Rumpf (19 %), das Retroperitoneum (15 %) sowie die Kopf-Hals-Region (9 %) [5, 6]. Die klinische Symptomatik ist variabel und abhängig von der Lokalisation und Größe des Tumors. Sie umfasst unklare, progrediente Weichteilschwellungen und -verhärtungen sowie die konsekutive Verdrängung, Kompression und Infiltration umliegender Strukturen mit entsprechender Klinik [5, 7]. Die Behandlung von Weichgewebesarkomen stellt eine interdisziplinäre Herausforderung dar, die eines spezialisierten Zentrums bedarf [8, 9]. Die Diagnostik und Therapie umfassen die radiologische Bildgebung, histopathologische Diagnosesicherung mittels Biopsie, das Staging sowie die Therapieplanung im interdisziplinären Tumorboard. Die Resektion stellt das Kernelement der Therapie adulter Weichteilsarkome dar. Die Residualtumorklassifikation (R-Klassifikation) bezeichnet den Resektionsstatus, dabei entspricht eine R0-Resektion mikroskopisch tumorfreien Resektionsrändern, wohingegen die R1- und R2-Resektion einen mikroskopischen respektive mikroskopische Residualtumor beschreibt. Entscheidend für eine kurative Therapie ist die R0-Resektion als weite Resektion [10], die als En-bloc-Resektion des Tumors inklusive des Biopsiezugangs durchgeführt werden sollte. Sekundäres Ziel ist ein extremitätenerhaltendes Vorgehen mit maximal möglichem Funktionserhalt.

Bei singulärer oder oligometastasierter Erkrankung anderer Tumorentitäten kann mitunter eine Resektion in Abhängigkeit vom onkologischen Gesamtkonzept in Erwägung gezogen werden.

Die Anatomie der Regio axillaris bedingt durch die Nähe zu den axillären Leitungsbahnen, insbesondere zum infraklavikulären Anteil des Plexus brachialis und den Vasa axillaria, die besondere Herausforderung der chirurgischen Resektion. Die R0-Resektion korreliert bei Sarkomen als entscheidender Parameter mit dem lokalrezidivfreien Gesamtüberleben [11, 12]. Darüber hinaus sind die Präparation der anatomischen Strukturen und die Schonung derselbigen entscheidend für den Funktionserhalt nach der operativen Resektion.

Die Entscheidung über das Ausmaß der chirurgischen Resektion und die Wahl des operativen Zugangsweges erfordert eine differenzierte Indikationsstellung basierend auf tumorbiologischen und patientenspezifischen Faktoren. Im Kontrast zur extremitätenerhaltenden Resektion ist die Amputation im Schultergürtel („forequarter amputation“) ein radikales und mutilierendes chirurgisches Vorgehen. Indikationen für eine „forequarter amputation“ umfassen ausgedehnte, aggressiv wachsende Weichgewebesarkome mit Infiltration neurovaskulärer Strukturen, der Thoraxwand oder des Schultergelenkes, Knochensarkome des Schultergürtels sowie ausgedehnte Tumorrezidive im Bereich des Schultergürtels [13–15].

Durch den Fortschritt in der Diagnostik, die Etablierung multimodaler Therapiekonzepte sowie die Verbesserung chirurgischer Techniken ist heutzutage in den meisten Fällen ein extremitätenerhaltendes Vorgehen möglich [13, 16, 17]. Hervorzuheben ist, dass in der Sarkomchirurgie das Ausmaß der Resektion und die Wahl des operativen Zugangsweges immer eine patientenindividuelle Entscheidung sind.

Die in diesem Artikel vorgestellte Operationstechnik beschreibt einen möglichen operativen Zugang für die Resektion in der Axilla lokalisierter Weichgewebesarkome sowie singulärer Metastasen anderer Tumorentitäten.

### Anatomie

Die Regio axillaris ist ein von Muskeln begrenzter Bindegewebsraum unterhalb der Fossa axillaris, deren Form einer vierseitigen Pyramide gleicht. Die Spitze der Pyramide projiziert sich etwa auf die Mitte der Clavicula, die Basis bildet die Fascia axillaris. Die ventrale Wand wird durch die Mm. pectorales major und minor und die Fascia clavipectoralis gebildet. Dorsalseitig sind die Mm. subscapularis, teres major und latissimus dorsi wandbildend. Die laterale Begrenzung bilden der proximale Humerus, der M. coracobrachialis und das Caput breve des M. biceps brachii.

#### Systematik der Leitungsbahnen der Regio axillaris.

Durch die Fossa axillaris ziehen die Leitungsbahnen der oberen Extremität, welche als Gefäß-Nerven-Strang im Bindegewebelager der Achselhöhle eingebettet sind. Chirurgische Eingriffe erfordern eine detaillierte Kenntnis dieser Leitungsbahnen, da die sorgfältige Präparation und Schonung derselbigen entscheidend für den Funktionserhalt sind.

#### Gefäße.

Die A. axillaris verläuft als Fortsetzung der A. subclavia distal der Clavicula durch den Axillarraum und schließlich an der medialen Seite des Humerusschafts. Sie wird begleitet von der medial liegenden V. axillaris. Die Vasa axillaria verlaufen in einer Gefäßscheide, die von den Ästen des Plexus brachialis umgeben ist.

Der *Plexus brachialis* (Rückenmarksegmente C5–Th1) innerviert die obere Extremität. Die Pars supraclavicularis besteht aus der Zusammenlagerung der Rami ventrales der Spinalnerven zu den Trunci superior, medius und inferior. Beim Übertritt in die Axilla erfolgt die Umlagerung zu den Fasciculi lateralis (C5–C7), posterior (C5–Th1) und medialis (C8–Th1). Von den Fasciculi der Pars infraclavicularis gehen die kurzen Äste zur Schultermuskulatur sowie die langen Endäste ab.

- Aus dem Fasciculus lateralis entspringt der *N. musculocutaneus*. Er verläuft medial der „conjoint tendons“ und innerviert den M. coracobrachialis und den Caput breve des M. biceps brachii.
- Der Hauptanteil des Fasciculus lateralis (Radix lateralis) vereinigt sich mit Fasern des Fasciculus medialis (Radix medialis) zum *N. medianus*. Distal der Medianusgabel verläuft der N. medianus an der Vorderseite der A. brachialis im Sulcus bicipitalis medialis zur Ellenbeuge.
- Der *N. ulnaris* entsteht aus dem Fasciculus medialis und verläuft medialseitig entlang der Gefäßscheide. Bedingt durch diese topografische Beziehung ist eine N.-ulnaris-Affektion durch Tumoren unterhalb des Plexus brachialis häufig, welche sich klinisch durch eine Kraftgradminderung oder neuropathische Schmerzen präsentieren kann.
- Der *N. radialis* verläuft als unmittelbare Fortsetzung des Fasciculus posterior dorsal der Gefäßscheide und zieht nach Verlassen der Regio axillaris zusammen mit der A. profunda brachii im Sulcus nervi ulnaris um den Humerus.
- Der *N. axillaris* verlässt den Fasciculus posterior und verläuft in der Tiefe der Regio axillaris unterhalb des Schultergelenks nach dorsal, um entlang des Collum chirurgicum auf die Hinterseite des proximalen Humerus zu gelangen.

#### Lymphbahnen.

Die Lymphknoten der Axilla sind wichtige Lymphknotenstationen für den Arm, den Schultergürtel sowie die vordere Brustwand. Größere Ansammlungen von Lymphknoten finden sich entlang der brachialen und axillären Gefäße, der lateralen thorakalen Gefäße und der subskapulären Gefäße. Die Lymphgefäße der Regio axillaris bilden den Plexus lymphaticus axillaris, welcher im Fettgewebe in enger topografischer Beziehung zur Gefäßscheide der A. und V. axillaris liegt.

## Operationsprinzip und -ziel

Das Operationsprinzip ist die weite Resektion des Tumors (Tab. 1). Die R0-Resektion stellt das Kernelement der Therapie adulter Weichgewebesarkome dar. Sekundäres Ziel ist ein extremitätenerhaltendes Vorgehen mit maximalem Funktionserhalt.

**Table 1:** 

Intraläsionale Resektion	Resektionsrand im Tumorgewebe
Marginale Resektion	Resektionsrand entlang der Pseudokapsel (reaktive Zone)
Weite Resektion	En-bloc-Resektion des Tumors und der reaktiven Zone mit einer umgebenden gesunden Gewebeschicht, Resektionsränder makroskopisch und mikroskopisch tumorfrei
Radikale Resektion	Vollständige Resektion des tumortragenden Kompartiments

## Vorteile

- Extremitätenerhaltendes Vorgehen mit maximal möglichem Funktionserhalt
- Wahrung der körperlichen Integrität durch den Extremitätenerhalt

## Nachteile

- Aufwendige Weichteilpräparation durch die Nähe zu den axillären Leitungsbahnen
- Gefahr der Gefäß- und Nervenverletzung
- Erhöhtes Risiko einer marginalen/intraläsionalen Resektion respektive Tumorperforation

## Indikationen

- Weichteilsarkome
- Singuläre Metastasen

## Kontraindikationen

- Fortgeschrittene Stadien mit Tumorinfiltration der neurovaskulären Strukturen, in denen ein extremitätenerhaltendes Vorgehen nicht möglich ist
- Lokalisation des Biopsiezugangs (Kontamination weiterer Kompartimente respektive der Gefäß‑/Nervenstraße) erfordert eine ausgedehnte Resektion
- Schlechter Allgemeinzustand mit eingeschränkter Operationsfähigkeit
- Ausgedehnte Metastasierung mit reduzierter Lebenserwartung (palliative Behandlungssituation)

## Patientenaufklärung

- Allgemeine Operationsrisiken
- Aufklärung über die Verletzung großer Gefäße mit entsprechendem Blutungsrisiko, Transfusionsbedarf, Notwendigkeit der Gefäßrekonstruktion, sekundäre Amputation
- Möglichkeit des Extremitätenverlusts bei nicht beherrschbaren Komplikationen
- Verletzung von Nerven des Plexus brachialis mit konsekutiven sensomotorischen Ausfällen
- Funktionseinschränkung der Extremität
- Intraoperativer Befund der Irresektabilität
- R1/R2-Resektion
- Lokalrezidiv

## Operationsvorbereitungen

Aufgrund der Seltenheit und Komplexität der Behandlung von Weichgewebesarkomen besteht die klare Empfehlung zur Diagnostik und Therapie in spezialisierten Zentren. Die Therapieplanung soll im interdisziplinären Tumorboard erfolgen (vertretene Fachdisziplinen: chirurgische Fachdisziplin mit dem Behandlungsschwerpunkt Weichteilsarkome, Hämatologie/Onkologie, Pathologie, Radiologie, Strahlentherapie, ggf. weitere Fachdisziplinen in Abhängigkeit der Lokalisation des Sarkoms).

**Figure 1:**
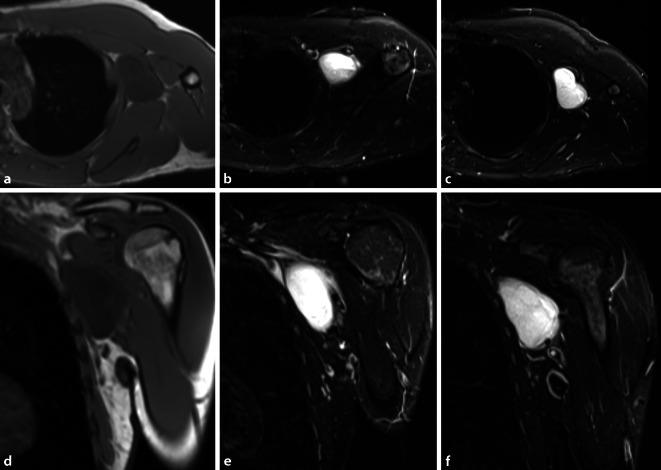

- Bildgebung
  - Bei klinischem Verdacht auf einen malignen Weichteiltumor ist die kontrastmittelverstärkte Magnetresonanztomographie mit Diffusionswichtung die bildgebende Methode der Wahl (Abb. 1).
- Bei Verdacht auf Gefäßaffektion durch den Tumor sollte präoperativ eine Gefäßdarstellung erfolgen.
- Biopsie
  - Die histologische Diagnosesicherung erfolgt mittels Biopsie (Stanzbiopsie, offene Biopsie) und sollte in einem spezialisierten Zentrum durchgeführt werden. Der Biopsiezugang soll im Bereich des späteren Operationszugangs liegen. Es ist der kürzeste, direkte Weg zum Tumor zu wählen, die vollständige Resektion des Biopsiekanals im Rahmen der Tumorresektion ist obligat. Die Planung der Lokalisation des Biopsiezugangs unter Berücksichtigung des operativen Zugangsweges sowie die Fadenmarkierung des Biopsiezugangs bei der Stanzbiopsie sind essenziell, damit die vollständige Resektion des Kanals ohne Erweiterung des Operationszugangs gewährleistet ist und ein extremitätenerhaltendes Vorgehen möglich ist.
  - Untersuchungen: histopathologische Subtypisierung, Graduierung, Molekularpathologie
- Staging
  - CT Thorax-Abdomen, Ausbreitungsdiagnostik je nach Tumorentität
- Präoperative Planung
  - Prätherapeutische Planung des Therapiekonzepts in der interdisziplinären Tumorkonferenz. Festlegung des Ausmaßes der Resektion in Abhängigkeit der Lokalisation, Tumorgröße, des Stagings sowie die Festlegung eines kurativen respektive palliativen Prozedere.
  - Planung der operativen Resektion sowie individuell erforderlicher rekonstruktiver Verfahren in einem interdisziplinären Ansatz. In Abhängigkeit des individuellen Befundes Hinzuziehen weiterer chirurgischer Fachabteilungen wie beispielsweise der Gefäßchirurgie, plastischen Chirurgie oder Thoraxchirurgie bei Beteiligung der Thoraxwand.
  - Die Resektion primärer Weichgewebesarkome der Extremitäten soll als weite Resektion erfolgen, die En-bloc-Resektion des Tumors inklusive des Biopsiezugangs ist anzustreben.
  - Präoperative klinische Untersuchung: Prüfung der N.-axillaris‑, N.-medianus‑, N.-radialis- und N.-ulnaris-Funktion (Sensibilität und Motorik).
  - Übliche präoperative Vorbereitung.
  - Bereitstellung einer ausreichenden Menge an Erythrozyten‑, Plasma- und Thrombozytenkonzentraten.

## Instrumentarium

- Gefäßsieb/Gefäßclips
- Feinchirurgische Instrumente
- Nervenstimulator
- Sehnennähte
- Bohrmaschine für transossäre Refixation
- Redondrainage

## Anästhesie und Lagerung

- Allgemeinanästhesie, perioperative Antibiotikaprophylaxe
- Lagerung: Rückenlage, Armtisch, ggf. Unterpolsterung zwischen den Schulterblättern
- Steriles Abwaschen und Abdeckung nach chirurgischem Standard

## Operationstechnik

**Figure 2:**
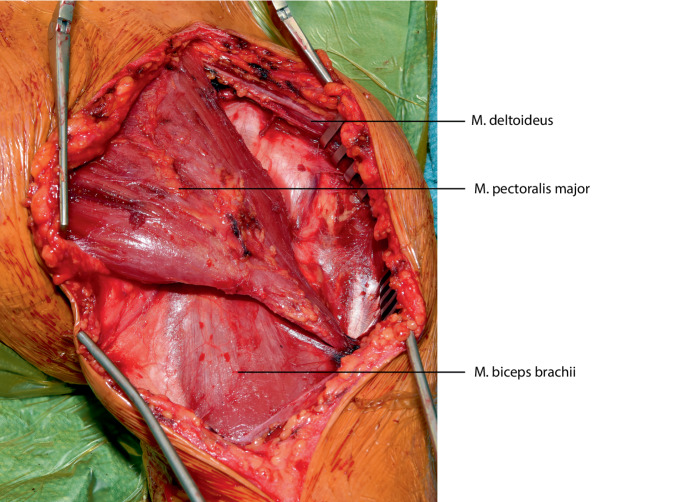

**Figure 3:**
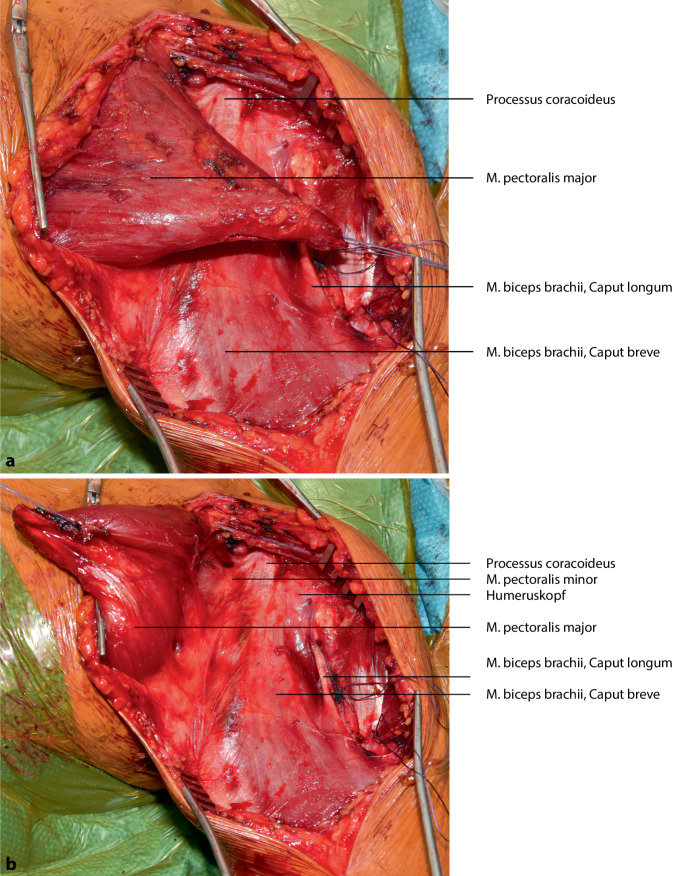

**Figure 4:**
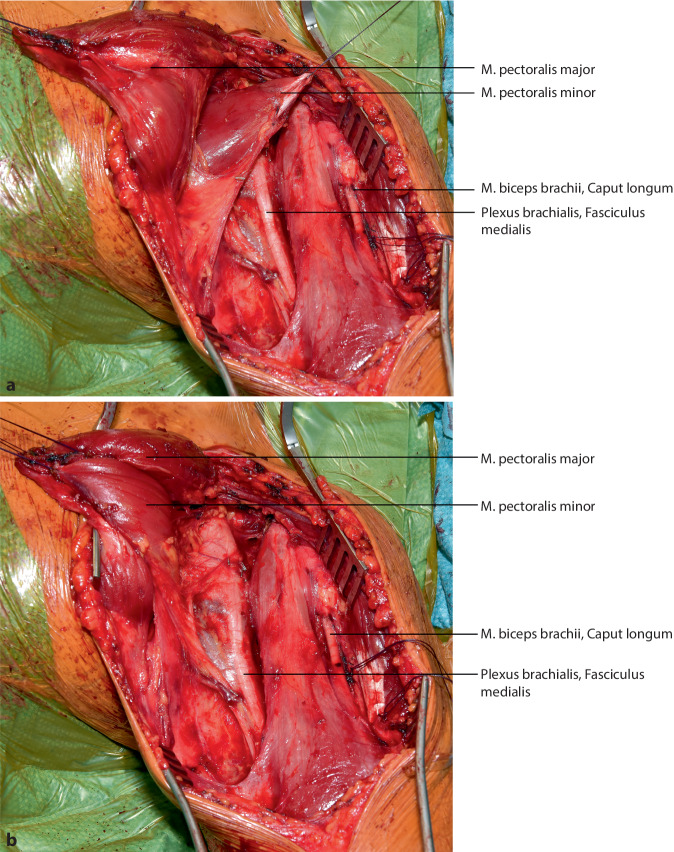

**Figure 5:**
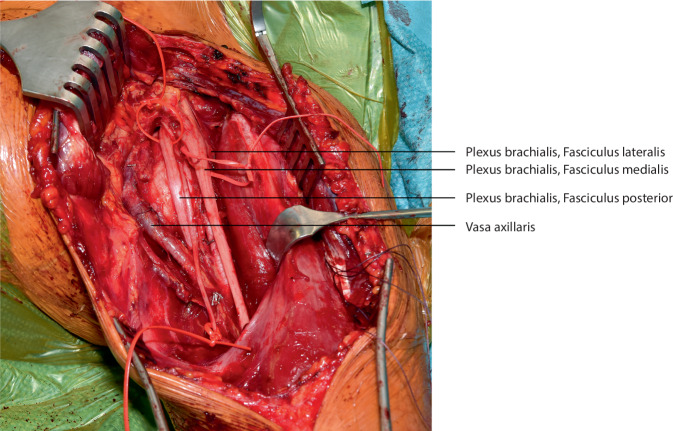

**Figure 6:**
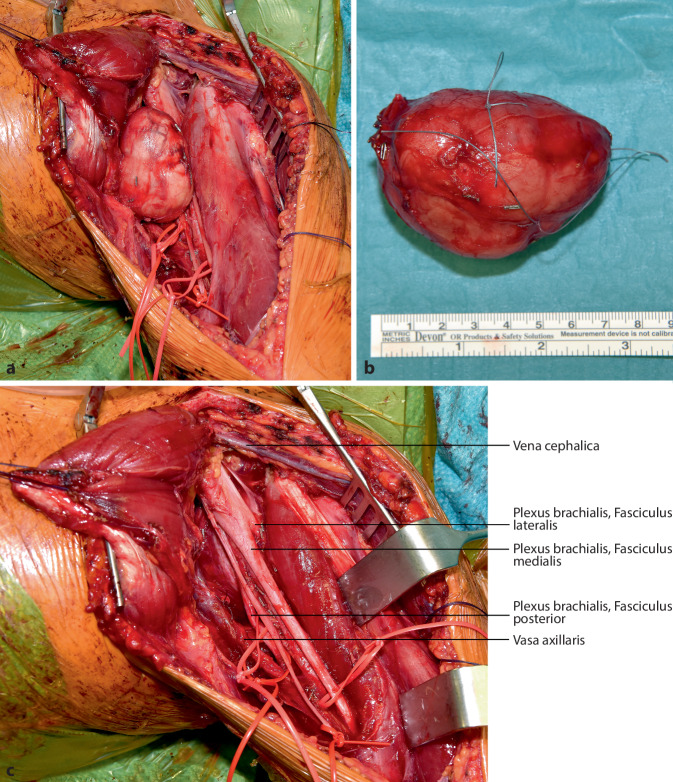

Die vorgestellte Operationstechnik beschreibt einen möglichen operativen Zugang zur Regio axillaris. Die Wahl des Operationszugangs im Rahmen onkologischer Resektionen ist dem individuellen Befund anzupassen. (Abb. 2, 3, 4, 5, 6, 7 und 8).

Anmerkung der Autoren: In dem gezeigten Fall wurde keine Biopsie durchgeführt, da es sich um die Resektion einer singulären Metastase eines Weichteilsarkoms in der Regio axillaris handelt.

**Figure 7:**
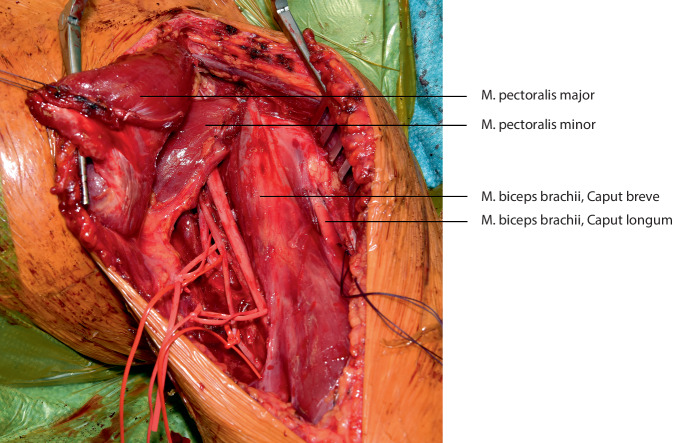

**Figure 8:**
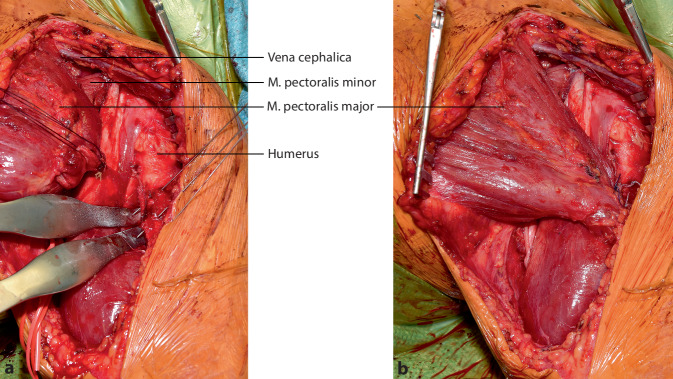

## Besonderheiten

**Figure 9:**
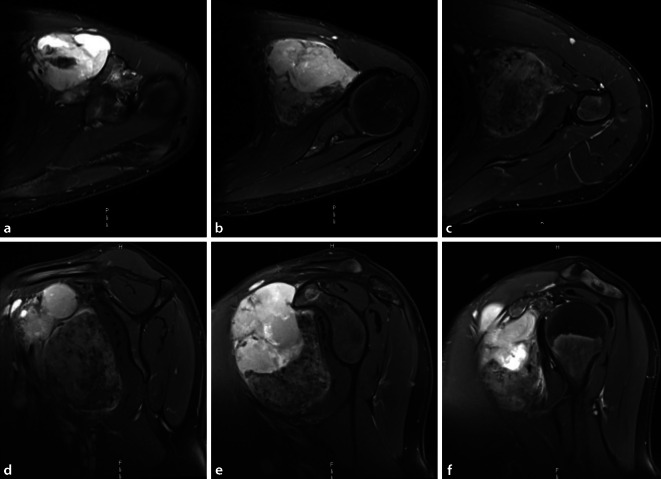

**Figure 10:**
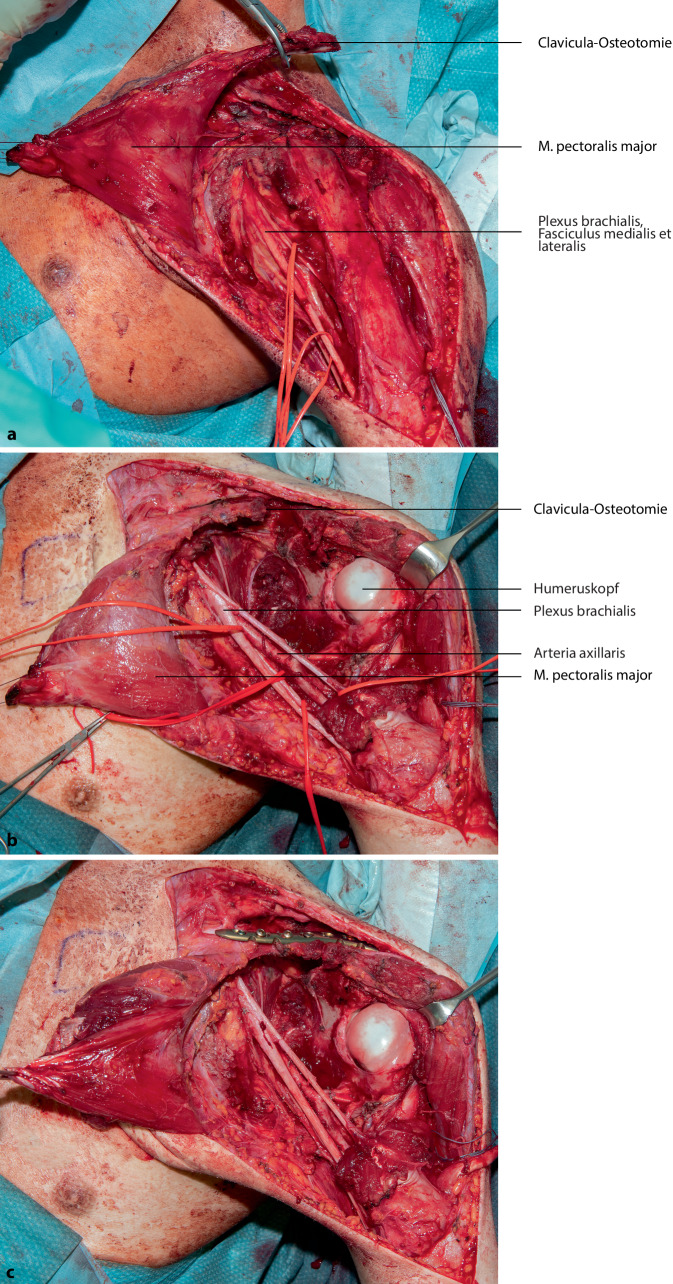

In Fällen mit einer Ausdehnung des Tumors bis zum Processus coracoideus kann eine Erweiterung der Operationstechnik durch die Durchführung einer Clavicula-Osteotomie zur besseren Übersicht und Darstellung der neurovaskulären Strukturen hilfreich sein (Abb. 9 und 10a, b). Im Anschluss ist die Rekonstruktion mittels Plattenosteosynthese erforderlich (Abb. 10c).

## Postoperative Behandlung

- Es erfolgt eine Drainagenanlage zur Vermeidung eines Seroms
- Anlage eines sterilen Wundverbands
- Elastokompressive Wicklung aufgrund der erforderlichen Resektion von Lymphbahnen
- Orthese: Anlage eines Schulterabduktionskissens für 6 Wochen, Kompressionsorthese
- Mobilisation: Woche 6 bis 12 passive Mobilisation, danach Beginn mit aktiver Mobilisation unter physiotherapeutischer Anleitung
- Onkologische Nachsorge (in dem vorgestellten Fall einer singulären Metastase eines myxoiden Liposarkoms erfolgte die adjuvante Radiatio)

## Fehler, Gefahren, Komplikationen

- Gefäßverletzung mit intraoperativem Blutverlust und Transfusionsbedarf: Gegebenenfalls ist bei dem intraoperativen Befund einer Gefäßarrosion ein Gefäßersatz notwendig.
- Nervenverletzung der großen Äste des Plexus brachialis: Dieses Risiko ist durch sorgfältige Präparation und den Einsatz eines Nervenstimulators zu verringern. Im Fall einer Nervenverletzung ist die Rekonstruktion mittels mikrochirurgischer Techniken zu erwägen. Bei nicht rekonstruierbarem Nervenschaden sind sekundär Muskelersatzplastiken zu erwägen.
- Ausbildung eines postoperativen Seroms: Gegebenenfalls ist eine operative Revision mit Ausschneidung der Seromhöhle notwendig.
- Postoperative Wundheilungsstörungen: Wundmanagement, frühzeitige Wundrevision bei Versagen der konservativen Therapie zur Vermeidung tiefer Wundinfektionen.
- Sekundäres Lymphödem der oberen Extremität: interdisziplinäre Nachbehandlung, ggf. Erwägung rekonstruktiver mikrochirurgischer Verfahren.
- Spezifische Komplikationen durch die adjuvante Bestrahlung der Achselhöhle (Lymphödem, funktionelle Beeinträchtigung, Wundprobleme).

## Ergebnisse

Die vorgestellten Daten wurden retrospektiv aus der Datenbank für muskuloskeletale Tumorerkrankungen an der Klinik für Unfallchirurgie der Medizinischen Hochschule Hannover erhoben. Im Zeitraum zwischen 2017 und 2022 erfolgte bei 6 konsekutiven Patienten die Resektion eines Tumors mit Lokalisation in der Regio axillaris. Die Diagnosen umfassten 4 primäre Weichteilsarkome (66,7 %) sowie 2 Metastasen in der Regio axillaris (33,3 %). Eine Patientin (16,7 %) war weiblich, das mittlere Alter zum Operationszeitpunkt betrug 58,7 Jahre. Das individuelle Therapieregime wurde in der interdisziplinären Tumorkonferenz festgelegt. In 3 Fällen eines primären Weichgewebesarkoms erfolgte die neoadjuvante Radiatio, in 1 Fall umfasste das multimodale Therapiekonzept die neoadjuvante Chemotherapie sowie adjuvante Strahlentherapie. Die Nachsorge erfolgte gemäß standardisierter Nachsorgeschemata in engmaschiger Anbindung in der Spezialsprechstunde für muskuloskeletale Tumorchirurgie. Das mittlere Follow-up lag bei 22,5 Monaten (3 bis 60 Monate).

Die chirurgische Resektion wurde in allen Fällen als weite Resektion durchgeführt. In 100 % der Fälle wurde eine primäre R0-Resektion erreicht. Es wurden keine Revisionseingriffe durchgeführt. Bezüglich des onkologischen Ergebnisses traten im Studienkollektiv keine Lokalrezidive auf.

Im Rahmen der Tumornachsorge wurde der Bewegungsumfang mittels Neutral-Null-Methode erhoben. Der durchschnittliche Bewegungsumfang für die Abduktion im Schultergelenk lag bei 135,0 ± 41,4° (Range 90–180°). In keinem Fall bestand postoperativ ein sensomotorisches Defizit.

Die subjektive Schulterfunktion („subjective shoulder value“ [SSV]) wird in % der gesunden Schulter angegeben und von dem Patienten selbst eingeschätzt [18]. Der SSV lag im Durchschnitt bei 80,0 ± 21,0 % (Range 50–100 %).

Das funktionale Ergebnis wurde mittels MSTS-Score (Musculoskeletal Society Tumour Score) erhoben. Der MSTS-Score basiert auf den Parametern Schmerz, Funktion und emotionaler Akzeptanz und bildet durch den erreichten Punktwert das funktionale postoperative Ergebnis ab (Enneking, Wada) [19, 20]. Die erreichte Anzahl wird zur maximal möglichen Punktzahl ins Verhältnis gesetzt und als prozentualer Wert angegeben. Hohe Werte entsprechen somit einer guten, niedrige Werte einer schlechten Funktion. Der mittlere MSTS-Score lag bei 89,5 % (Range 32–100 %) und belegt somit ein gutes funktionales Outcome im Studienkollektiv.
